# RETREG3/FAM134C phosphorylation by CSNK2 regulates reticulophagy during starvation

**DOI:** 10.1080/27694127.2022.2131212

**Published:** 2022-10-06

**Authors:** Giorgia Di Lorenzo, Francescopaolo Iavarone, Marianna Maddaluno, Paolo Grumati, Carmine Settembre

**Affiliations:** a Telethon Institute of Genetics and Medicine (TIGEM), Pozzuoli, Italy; b Department of Clinical Medicine and Surgery, Federico II University, Naples, Italy

**Keywords:** CSNK2, endoplasmic reticulum, MTORC1, reticulophagy, RETREG3

## Abstract

Starvation is the most potent physiological inducer of autophagy, the catabolic process which degrades unessential cytosolic components to sustain cellular homeostasis and survival. During starvation, the mechanisms of autophagy activation have been extensively investigated; however, less is known about how substrate selection occurs. In this punctum, we summarize our recent findings that delineate a novel signaling pathway that promotes selective autophagic removal of parts of the endoplasmic reticulum (reticulophagy) during starvation. We demonstrate that the inhibition of MTORC1 results in the activation of the reticulophagy receptor RETREG3/FAM134C by preventing its phosphorylation by CSNK2/CK2. *In vivo*, RETREG3 depletion impairs MTORC1-dependent regulation of lipid metabolism in liver. Last, we describe a novel approach to study selective autophagy *in vivo*, which might be exploited to identify novel physiological roles of autophagy.

To overcome starvation or other stress conditions, cells activate macroautophagy/autophagy, a catabolic process required for the degradation and recycling of cellular components by lysosomes. MTOR (mechanistic target of rapamycin kinase) complex 1 (MTORC1) is an important nutrient sensor and regulator of autophagy. Starvation inhibits MTOR activity leading to the activation of the autophagy machinery that is deputed to autophagosome biogenesis. Substrates (organelles, protein complexes or lipids) are engulfed within autophagosomes thanks to the activity of autophagy receptors that stably or transiently associate with the substrate(s) and bind to LC3 or GABARAP autophagic membrane proteins, through the LC3-interating region (LIR) or GABARAP-interacting motif (GIM), respectively. How substrate recruitment is regulated during autophagy induction is currently an important question in the field.

We specifically studied selective removal of part of the endoplasmic reticulum by autophagy (reticulophagy), which is activated by starvation and is emerging as an important ER quality control pathway. Reticulophagy receptors drive the engulfment of ER fragments by phagophores. Among them, the ER membrane proteins RETREG2/FAM134A and RETREG3 were recently characterized and together with RETREG1/FAM134B, share structural similarities including the reticulum homology and LIR domains which allow them to bend the ER membrane and bind to LC3, respectively. Whether RETREG proteins respond to different stimuli remains to be clarified.

In our recent study [1] we generated novel tools to monitor reticulophagy *in vivo*, using an adeno-associated virus (AAV) expressing the tandem reporter ssRFP-GFP fused to the ER marker KDEL. We injected wild-type mice with AAV2/9-ss-RFP-GFP-KDEL and observed that upon starvation there is a significant induction of reticulophagy in the liver. This induction is dependent on MTOR being inhibited, as it is completely blunted in mice with constitutively active MTOR signaling. Among RETREG proteins, RETREG3 levels are the most influenced by MTOR signaling, suggesting a primary role of RETREG3 during MTOR-induced reticulophagy. Notably in contrast to RETREG1, whose levels increase, RETREG3 is quickly degraded in mouse liver upon MTOR inhibition, suggesting that these two proteins are differentially regulated by MTOR.

Next, we investigated the RETREG3 activation mechanism during starvation. We performed a phospho-proteomic analysis and identified different phospho-sites on the RETREG3 protein that are modulated during starvation. Three residues (S435, S436 and T440), located in proximity of the LIR domain (-12, -11 and -7, respectively), caught our attention because they might be implicated in RETREG3 binding to LC3 proteins. Indeed, *in silico* studies suggest that these residues interact with a neutrally charged region of LC3B; hence, phosphorylation, increasing the local electron density, may weaken the RETREG3-LC3B interaction. Consistently, *in vitro* and *in vivo* experiments demonstrate that phosphorylated RETREG3 shows a reduced affinity for LC3B and an impaired lysosomal delivery upon MTOR inhibition. In summary, these data suggest that phosphorylation regulates RETREG3 activation.

The S435, S436 and T440 residues show a consensus motif for CSNK2/CK2 (casein kinase 2). Indeed, through an *in vitro* kinase assay, CSNK2 pharmacological inhibition and co-immunoprecipitation experiments, we demonstrated that RETREG3 is a novel CSNK2 substrate. CSNK2 inhibition results in a dephosphorylated RETREG3, which in turn enhances the interaction with LC3B and lysosomal degradation. CSNK2 is a tetrameric kinase complex formed by the assembly of two catalytic (⍺ or ⍺′) with two regulatory (β) subunits. Notably, we observed that genetic and pharmacological inhibition of MTORC1 reduces the phosphorylation levels of several CSNK2 substrates, including RETREG3. This effect might be due to a direct phosphorylation of CSNK2B/CK2β by MTORC1.

The involvement of CSNK2 kinase in autophagy has been previously observed. CSNK2 phosphorylates the mitophagy receptor FUNDC1 and the reticulophagy receptor TEX264, reducing or enhancing, respectively, the binding affinity for the LC3/GABARAP proteins. The explanation for these opposite effects can be found by the position of phosphorylated residues. In the case of RETREG3 and FUNDC1, CSNK2 phosphorylates residues that are predicted to interact with a neutrally charged region of LC3B (Figure 1). Conversely, phospho-sites in TEX264 are adjacent to the LIR motif, that binds the positively charged LC3B pocket (Figure 1). Thus, the same kinase might have antagonistic roles on autophagy receptors causing paradoxically opposite effects. Consistently, we found that CSNK2 inhibition induces reticulophagy in cells that express high levels of endogenous RETREG3, such as A549 alveolar cells, while it has no appreciable effect in cells that express lower levels of RETREG3 like U2OS cells. These data suggest that the expression levels of autophagy receptors define autophagy cargo selectivity in a cell- and tissue-specific manner.

**Figure 1.**
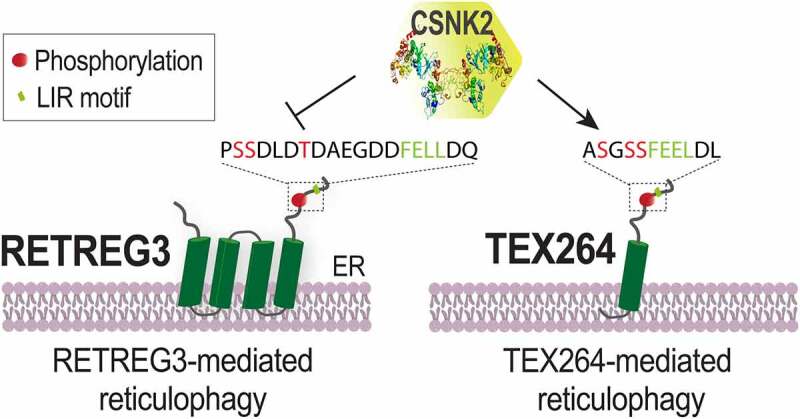
Proposed model of reticulophagy regulation by CSNK2. CSNK2 phosphorylates the reticulophagy receptors RETREG3 and TEX264 at the indicated residues (red) promoting or inhibiting, respectively the binding of the LIR domain (green) with LC3.

The observations that, among RETREG paralogs, RETREG3 has a more prominent role in mediating MTOR-dependent reticulophagy, prompted us to search for a role of RETREG3 in MTORC1-mediated regulation of metabolism. We observed abnormal lipid accumulation in the liver of *retreg3* knockout (KO) mice in which MTOR signaling is pharmacologically inhibited. This phenotype is not present in the liver of *retreg1* KO mice, revealing physiologically distinct functions of RETREG proteins, at least in liver. How RETREG3 controls liver lipid metabolism needs to be investigated. By quantitative proteomic analysis using cell lines lacking RETREG proteins, we observed that RETREG3 is required for the degradation of ER-localized lipid biosynthetic enzymes during MTORC1 inhibition, suggesting that RETREG3-mediated reticulophagy can limit lipid synthesis by the ER.

Overall, our work describes a new mechanism driving substrate selection during starvation-induced autophagy, suggesting a link between the metabolic and quality control functions of autophagy that might be exploited for therapeutic purposes.
